# Supplementary material for: Multi‐dataset identification of innovative feature genes and molecular mechanisms in keratoconus
Source: J Cell Mol Med. 2024 Sep 19;28(18):e70079. doi: 10.1111/jcmm.70079 (PMC11412914; doi:10.1111/jcmm.70079)
Supplement: Supplementary file 2 — Material S2. [file JCMM-28-e70079-s002.zip › 7.GSEA analysis/4.MYRF/KEGG_P53_SIGNALING_PATHWAY.html]

Details for gene set KEGG\_P53\_SIGNALING\_PATHWAY[GSEA]

|  || Dataset | MYRF.group.cls#h\_versus\_l |
| Phenotype | group.cls#h\_versus\_l |
| Upregulated in class | l |
| GeneSet | KEGG\_P53\_SIGNALING\_PATHWAY |
| Enrichment Score (ES) | -0.39918822 |
| Normalized Enrichment Score (NES) | -1.4857132 |
| Nominal p-value | 0.07966457 |
| FDR q-value | 0.20426585 |
| FWER p-Value | 0.776 |
Table: GSEA Results Summary

  

Fig 1: Enrichment plot: KEGG\_P53\_SIGNALING\_PATHWAY      
 Profile of the Running ES Score & Positions of GeneSet Members on the Rank Ordered List

  

| SYMBOL | TITLE | RANK IN GENE LIST | RANK METRIC SCORE | RUNNING ES | CORE ENRICHMENT || 1 | CCNE2 | na | 2609 | 0.317 | -0.0400 | No |
| 2 | APAF1 | na | 5026 | 0.221 | -0.0850 | No |
| 3 | TSC2 | na | 7828 | 0.142 | -0.1499 | No |
| 4 | TP53AIP1 | na | 8006 | 0.138 | -0.1396 | No |
| 5 | ATM | na | 8036 | 0.138 | -0.1252 | No |
| 6 | IGF1 | na | 8743 | 0.121 | -0.1320 | No |
| 7 | CASP3 | na | 8824 | 0.120 | -0.1210 | No |
| 8 | ATR | na | 9220 | 0.111 | -0.1200 | No |
| 9 | CASP8 | na | 9334 | 0.109 | -0.1111 | No |
| 10 | MDM4 | na | 10503 | 0.087 | -0.1352 | No |
| 11 | GADD45G | na | 10627 | 0.084 | -0.1293 | No |
| 12 | SHISA5 | na | 10684 | 0.083 | -0.1217 | No |
| 13 | PTEN | na | 11228 | 0.075 | -0.1290 | No |
| 14 | CCNE1 | na | 11231 | 0.075 | -0.1208 | No |
| 15 | CDK1 | na | 11292 | 0.073 | -0.1143 | No |
| 16 | SESN1 | na | 12406 | 0.058 | -0.1400 | No |
| 17 | TP53 | na | 12977 | 0.051 | -0.1508 | No |
| 18 | RCHY1 | na | 13241 | 0.047 | -0.1532 | No |
| 19 | BID | na | 13268 | 0.047 | -0.1487 | No |
| 20 | MDM2 | na | 13578 | 0.043 | -0.1529 | No |
| 21 | PPM1D | na | 14443 | 0.033 | -0.1741 | No |
| 22 | STEAP3 | na | 14607 | 0.031 | -0.1753 | No |
| 23 | CCND1 | na | 14657 | 0.031 | -0.1733 | No |
| 24 | CD82 | na | 14673 | 0.031 | -0.1703 | No |
| 25 | DDB2 | na | 15515 | 0.023 | -0.1920 | No |
| 26 | PIDD1 | na | 16292 | 0.015 | -0.2128 | No |
| 27 | TP73 | na | 16394 | 0.014 | -0.2142 | No |
| 28 | TP53I3 | na | 16682 | 0.011 | -0.2212 | No |
| 29 | BAX | na | 17915 | 0.000 | -0.2567 | No |
| 30 | PERP | na | 18504 | -0.005 | -0.2731 | No |
| 31 | CDKN2A | na | 19779 | -0.019 | -0.3077 | No |
| 32 | CCNG1 | na | 19788 | -0.019 | -0.3058 | No |
| 33 | SERPINB5 | na | 19986 | -0.022 | -0.3091 | No |
| 34 | THBS1 | na | 22189 | -0.048 | -0.3672 | No |
| 35 | SFN | na | 22799 | -0.057 | -0.3785 | No |
| 36 | CASP9 | na | 23052 | -0.060 | -0.3790 | No |
| 37 | CYCS | na | 23572 | -0.069 | -0.3864 | No |
| 38 | CCND3 | na | 23579 | -0.069 | -0.3789 | No |
| 39 | CCNB3 | na | 24284 | -0.080 | -0.3902 | Yes |
| 40 | EI24 | na | 24337 | -0.081 | -0.3827 | Yes |
| 41 | SIAH1 | na | 24671 | -0.088 | -0.3826 | Yes |
| 42 | CDK4 | na | 24674 | -0.088 | -0.3729 | Yes |
| 43 | CCNB2 | na | 25069 | -0.094 | -0.3737 | Yes |
| 44 | ZMAT3 | na | 25327 | -0.099 | -0.3701 | Yes |
| 45 | TNFRSF10B | na | 25657 | -0.105 | -0.3678 | Yes |
| 46 | CDK6 | na | 25789 | -0.108 | -0.3595 | Yes |
| 47 | CCND2 | na | 25792 | -0.109 | -0.3475 | Yes |
| 48 | SESN2 | na | 25869 | -0.110 | -0.3375 | Yes |
| 49 | GTSE1 | na | 26206 | -0.117 | -0.3341 | Yes |
| 50 | CCNB1 | na | 26279 | -0.119 | -0.3229 | Yes |
| 51 | RRM2B | na | 26864 | -0.132 | -0.3251 | Yes |
| 52 | CHEK2 | na | 27601 | -0.151 | -0.3296 | Yes |
| 53 | GADD45B | na | 27736 | -0.154 | -0.3163 | Yes |
| 54 | FAS | na | 28092 | -0.163 | -0.3084 | Yes |
| 55 | CDK2 | na | 29418 | -0.203 | -0.3240 | Yes |
| 56 | PMAIP1 | na | 30235 | -0.232 | -0.3217 | Yes |
| 57 | BBC3 | na | 30993 | -0.263 | -0.3143 | Yes |
| 58 | CCNG2 | na | 31062 | -0.267 | -0.2865 | Yes |
| 59 | CHEK1 | na | 31328 | -0.281 | -0.2628 | Yes |
| 60 | RPRM | na | 32117 | -0.324 | -0.2494 | Yes |
| 61 | GADD45A | na | 32736 | -0.368 | -0.2263 | Yes |
| 62 | SESN3 | na | 33022 | -0.389 | -0.1912 | Yes |
| 63 | RRM2 | na | 33541 | -0.449 | -0.1562 | Yes |
| 64 | CDKN1A | na | 33771 | -0.483 | -0.1091 | Yes |
| 65 | IGFBP3 | na | 33925 | -0.508 | -0.0569 | Yes |
| 66 | SERPINE1 | na | 34570 | -0.719 | 0.0046 | Yes |
Table: GSEA details [plain text format]

  

Fig 2: KEGG\_P53\_SIGNALING\_PATHWAY      
 Blue-Pink O' Gram in the Space of the Analyzed GeneSet

  

Fig 3: KEGG\_P53\_SIGNALING\_PATHWAY: Random ES distribution      
 Gene set null distribution of ES for **KEGG\_P53\_SIGNALING\_PATHWAY**
